# Synthesis and stability of strongly acidic benzamide derivatives

**DOI:** 10.3762/bjoc.14.38

**Published:** 2018-02-27

**Authors:** Frederik Diness, Niels J Bjerrum, Mikael Begtrup

**Affiliations:** 1 Department of Drug Design and Pharmacology, University of Copenhagen, Universitetsparken 2, DK-2100 Copenhagen, Denmark; 2 Present address: Department of Chemistry, University of Copenhagen, Universitetsparken 5, DK-2100 Copenhagen, Denmark; 3 Department of Energy Conversion and Storage, Technical University of Denmark, Kemitorvet, Building 207, room 042, DK-2800 Kgs. Lyngby, Denmark

**Keywords:** benzoic acid, cross-coupling, hydrolysis, S_N_Ar, trifluoromethanesulfonamide

## Abstract

Reactivity studies of strong organic acids based on the replacement of one or both of the oxygens in benzoic acids with the trifluoromethanesulfonamide group are reported. Novel derivatives of these types of acids were synthesized in good yields. The generated *N*-triflylbenzamides were further functionalized through cross-coupling and nucleophilic aromatic substitution reactions. All compounds were stable in dilute aqueous solutions. Studies of stability under acidic and basic conditions are also reported.

## Introduction

**Figure 1:**
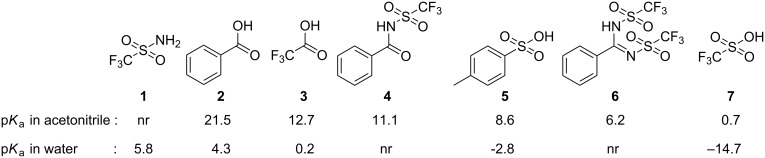
Acid strength (p*K*a) of various organic acids in acetonitrile or water (nr = not reported) [12–14].

Very strong organic acids are interesting as catalysts for chemical reactions [1–2] and for facilitation of proton conduction [3]. In order to enable their incorporation into functional materials (e.g., polymers), these acids need additional functionality in the form of a reactive group, and the chemistry applied for functionalization must be compatible with their strong acidic nature. Many types of strong organic acids such as triflic acid (**7**) or trifluoroacetic acid (**3**) are not readily modified, and changing substituents on these acids will heavily impact their p*K*_a_ value (Figure 1). In contrast, halogenated benzoic acid derivatives are easily functionalized through cross-coupling [4–5] or nucleophilic aromatic substitution reactions (S_N_Ar) [6–7]. Benzoic acids (e.g., **2**) are relatively weak acids, even with highly electron-withdrawing substituents on the aromatic core [8]. Very strong benzoic acid derivatives (e.g., **4** and **6**) have been synthesized by replacing one or both of the oxygens of the carboxylate group with the trifluoromethanesulfonamide (**1**) group [9–11].

**Figure 2:**
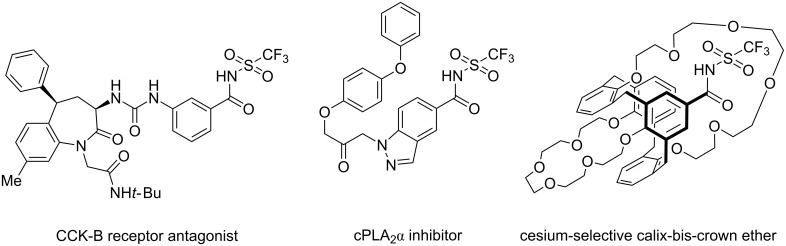
Examples of functional molecules containing an *N*-triflylbenzamide.

Interestingly, these types of compounds have attracted little attention and have not thoroughly been explored with regards to their applications. Few publications report the application of *N*-triflylbenzamides as benzoic acid bioisosteres in receptor antagonists and enzyme inhibitors (Figure 2) [15–18]. The reported derivatives all displayed activity, but only with similar or reduced potency compared to the corresponding benzoic acid derivatives. Application of deprotonated *N*-triflylbenzamide derivatives as counter anions in supramolecular crown ether compounds for metal ion extraction has also been reported by one group (Figure 2) [19–25]. All the *N*-triflylbenzamide constructs generated for this purpose have proved superior to the corresponding benzoate derivatives with regards to metal ion extraction capability.

**Scheme 1:**
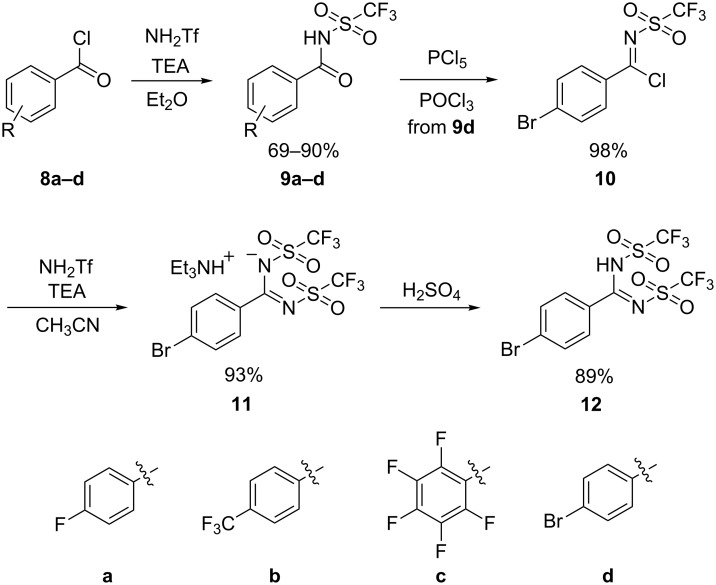
Synthesis of the strongly acidic benzamide derivatives.

The *N*-triflylbenzamides are simple to generate by the reaction between trifluoromethanesulfonamide (**1**) and an activated benzoyl derivative such as benzoyl chlorides (e.g., **8a**, Scheme 1) [9,15]. The reactions are generally high yielding and the products are simple to isolate in their protonated form by recrystallization. The generation of *N*-triflylbenzamides by direct reaction of various triflyl derivatives with benzamides or benzoic acids has been less systematically explored. Most compounds synthesized by these approaches are N-alkylated or N-arylated and have been formed unintentionally as byproducts [26–33]. Finally, two examples of syntheses via palladium-catalyzed carbonylation of trifluoromethanesulfonamide (**1**) have been reported [34–35]. The *N*-triflylbenzamides have only been explored as substrates in a few reactions. A recent report describes using *N*-triflylbenzamide as a directing group in a ruthenium-catalyzed C–H activation reaction [36]. Most other reported transformations pass via the imidoyl chloride intermediates (equivalent to **10**) to the amidine derivatives, which have been used in rearrangement reaction studies [9,37–39]. By these means also *N*,*N*’-bis(triflyl)benzimidamides (also termed benzamidines) have been generated, but only one report describes their syntheses [10]. The *N*-triflylbenzamides are stronger acids than any of the carboxylic acids, including trifluoroacetic acid (**3**). The *N*,*N*’-bis(triflyl)benzimidamides are very strong organic acids, much stronger than *p*-toluenesulfonic acid (**5**) which is commonly used as a soluble organic acid catalyst in chemical reactions. Remarkably, neither the *N*-triflylbenzamides nor the *N*,*N*’-bis(triflyl)benzimidamides have been studied as Brønsted acid catalysts. Their chemical stability including compatibility with conditions applied to common chemical transformations has not been described. With the prospect of using these strong benzoic acid derivatives for enhancing proton conductivity in proton-exchange membrane (PEM) fuel cells [3,40] we have examined their compatibility with chemical transformations as well as their stability towards hydrolytic conditions.

## Results and Discussion

In these studies, four substituted *N*-triflylbenzamides **9a**–**d** were synthesized by the reaction of the corresponding benzoyl chloride with trifluoromethanesulfonamide (**1,** Scheme 1). The known 4-fluoro-*N*-triflylbenzamide (**9a**) was synthesized according to the previously reported method [9] and with few adjustments this methodology was also applied for the generation of three new derivatives **9b**–**d**, which were obtained in good yields. The 4-bromo derivative **9d** was further converted into the *N*,*N*’-bis(triflyl)benzimidamide **12** by formation of the corresponding imidoyl chlorides **10** with PCl_5_ in POCl_3_, followed by the additional reaction with trifluoromethanesulfonamide (**1**) and protonation by sulfuric acid (Scheme 1) [10].

**Scheme 2:**
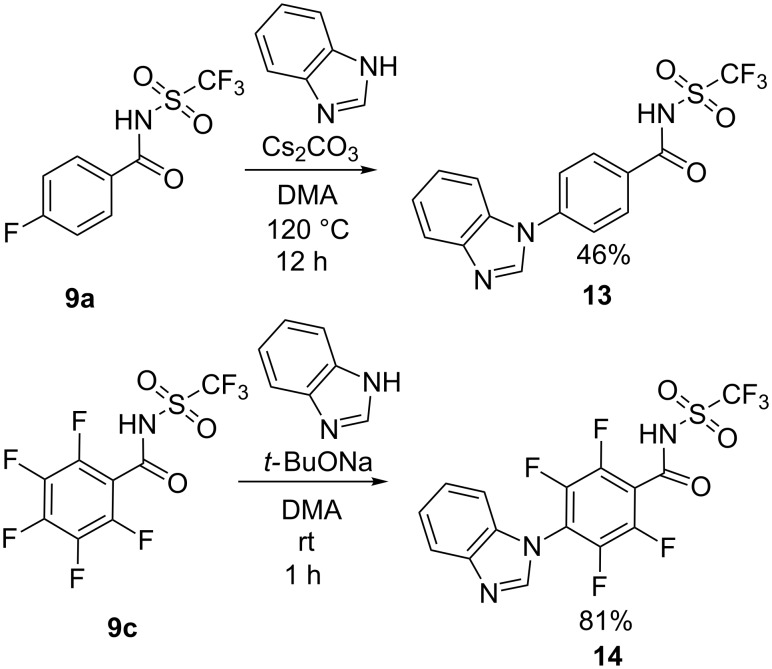
S_N_Ar reactions of fluoro-substituted benzamide derivatives.

In recent years, we have reported high yielding catalyst-free N-arylation by S_N_Ar reaction of mono- or perfluorobenzene derivatives [41–43]. Hence, it was proposed that the 4-fluoro and the pentafluorobenzamide derivatives **9a** and **9c** could be functionalized through S_N_Ar reactions. Thus, compounds **9a** and **9c** were reacted with benzimidazole under previously developed conditions (Scheme 2) [41–42]. The benzimidazole was chosen as model nucleophile for polybenzimidazole, a polymer commonly applied as a membrane in PEM fuel cells [40]. These reactions provided the 4-benzimidazolyl derivatives **13** and **14** in good yields and the *N*-triflylbenzamide group proved to be stable under these reaction conditions. Gratifyingly it was possible to perform selective mono-substitution of the pentafluoro derivative regioselectively in the 4-position, affording only compound **14**. This is in line with previous observations of S_N_Ar reactions on pentafluorobenzene derivatives [42]. Due to the zwitterionic nature of the products, reversed-phase chromatography was chosen to simplify the purifications.

**Scheme 3:**
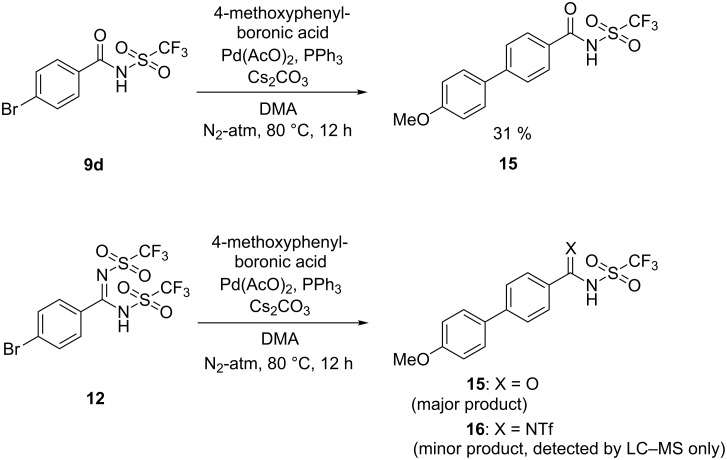
Cross-coupling reactions of *N*-triflylbenzoic acid derivatives.

The *N*-triflylbenzamide group also proved stable to cross-coupling reaction conditions, as exemplified by a palladium-catalyzed Suzuki–Miyaura reaction of the 4-bromo-substituted derivative **9d** (Scheme 3). The corresponding *N*,*N*’-bis(triflyl)benzimidamide derivative **12** was also tested under identical conditions. The reaction proceeded with high conversion, but surprisingly the major product of this reaction was also the *N*-triflylbenzamide **15**. Additional experiments revealed that the *N*,*N*’-bis(triflyl)benzimidamide group was unstable in aqueous basic conditions.

**Scheme 4:**
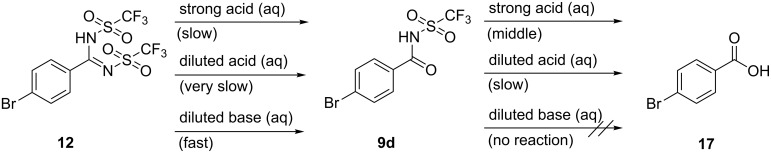
Hydrolysis rates of the 4-bromobenzoic acid derivatives.

This led to concerns about the stability of the products in general. In addition, during characterization by NMR it was also noted that some samples of the *N*-triflylbenzamide products contained small amounts of the parent benzoic acid, despite prior purification by recrystallization. Further studies revealed that the content of benzoic acid increased over time in the solutions and the conversion rate was concentration dependent, the reason being simple hydrolysis auto-catalyzed by the acids themselves. This phenomenon was also observed for *N*,*N*’-bis(triflyl)benzimidamide **12** but at slower rate. Hence, an elaborated study of the products’ stability towards acid or base promoted hydrolysis was undertaken (Scheme 4). Dilute aqueous solutions (0.5 mg/mL) of the *N*-triflylbenzamides displayed no sign of degeneration even after 24 h. The compounds also remained fully intact in 0.5 M aqueous NaOH. In solutions of 0.5 M aqueous HCl the compounds very slowly degraded over weeks. The *N*,*N*’-bis(triflyl)benzimidamide **12** was also stable in dilute aqueous solutions and was even more stable in 0.5 M aqueous HCl than the corresponding *N*-triflylbenzamide **9d**. Hence, in the acid-catalyzed hydrolysis reaction of **12** the 4-bromo-*N*-triflylbenzamide (**9d**) was only detected as a trace as **9d** converted faster to 4-bromobenzoic acid than compound **12** converted to **9d**. In contrast, 0.5 M aqueous NaOH rapidly hydrolyzed the *N*,*N*’-bis(triflyl)benzimidamide **12** to yield the base-stable *N*-triflylbenzamide **9d**.

**Figure 3:**
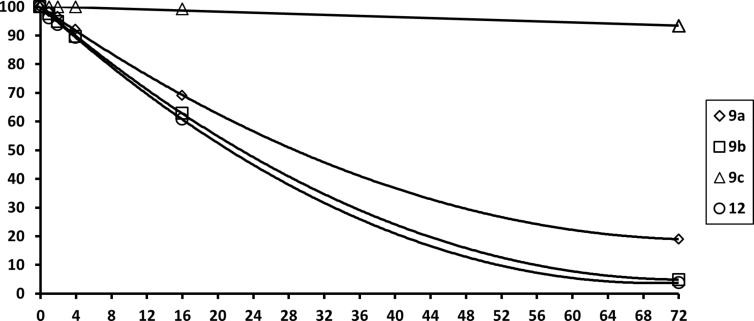
Content (percent) of super acids (0.5 mg/mL) over time (hours) in H_3_PO_4_/H_2_O/MeOH 17:3:20 at 50 °C.

Neither the mono-substituted *N*-triflylbenzamides **9a** and **9b** nor the 4-bromo-*N*,*N*’-bis(triflyl)benzimidamide (**12**) were stable in heated methanolic phosphoric acid, which is commonly used for operating PEM fuel cells (Figure 3) [44]. However, most surprisingly, the pentafluoro *N*-triflylbenzamide (**9c**) was found to be much more stable under these conditions, which is promising for future applications in proton conducting materials.

## Conclusion

In summary, it has been demonstrated that novel mono- or bis-trifluoromethanesulfonamide derivatives of benzoic acids bearing a reactive group (bromine or fluorine) may be generated in good yields and further functionalized through nucleophilic aromatic substitution or cross-coupling reactions. It was found that the products were stable in dilute aqueous solutions, but they slowly hydrolyzed in concentrated solutions or in the presence of other strong acids. The *N*-triflylbenzamides **9a**–**d** were stable in basic aqueous solutions whereas the tested bromo-*N*,*N*’-bis(triflyl)benzimidamide **12** rapidly hydrolyzed to the corresponding bromo-*N*-triflylbenzamide **9d**. Under conditions simulating ambient PEM fuel cells operation the 4-substituted benzamide derivatives **9a**,**b**,**d** and **12** were about 50% degraded within 24 h, whereas the pentafluoro *N*-triflylbenzamide (**9c**) under the same conditions was less than 5% degraded.

## Experimental

### General aspects

All purchased chemicals were used without further purification. All solvents were HPLC grade. NMR data were recorded on a Bruker 500 MHz spectrometer at 298 K with methanol-*d*_4,_ DMSO-*d*_6_ or hexafluorobenzene as internal standard. High-resolution mass spectrometry (HRMS) was performed on a Bruker MALDI–TOF spectrometer.

### General procedure

Benzoyl chloride (10.0 mmol) in Et_2_O (10 mL) was added dropwise to a solution of trifluoromethanesulfonamide (1.5 g, 10.0 mmol) and triethylamine (3.5 mL, 25 mmol) in Et_2_O (30 mL) at 0 °C over 10 min. The mixture was stirred for 30 min and then heated to reflux for 2 h. The reaction was filtered and the solvent was removed from the filtrate. The crude product was mixed with 50% H_2_SO_4_ (aq, 50 mL) and the mixture stirred for 30 min. The precipitate was isolated and dried overnight. The product was purified by recrystallization from toluene.

**4-Fluoro-*N*-((trifluoromethyl)sulfonyl)benzamide (9a)** [9]: Synthesized by general procedure. The product was obtained as small clear crystalline flakes (1.88 g, 69%). mp 152–154 °C; ^1^H NMR (500 MHz, methanol-*d*_4_) δ 7.99 (dd, *J* = 9.0, 5.2 Hz, 2H), 7.29 (t, *J* = 8.8 Hz, 2H); ^13^C NMR (126 MHz, methanol-*d*_4_) δ 167.6 (d, *J* = 254.3 Hz), 166.0, 132.8 (d, *J* = 9.6 Hz), 129.1 (d, *J* = 3.1 Hz), 121.0 (q, *J* = 321.5 Hz), 117.1 (d, *J* = 22.7 Hz); ^19^F NMR (470 MHz, methanol-*d*_4_) δ −76.93 (s, 3F), −105.75 (s, 1F); HRMS (TOF) *m*/*z*: [M − H]^−^ calcd for C_8_H_4_F_4_NO_3_S^−^, 269.9854; found, 269.9888.

**4-Trifluoromethyl-*N*-((trifluoromethyl)sulfonyl)benzamide (9b):** Synthesized by general procedure. The product was obtained as white powder (2.83 g, 88%). mp 174–179 °C; ^1^H NMR (500 MHz, methanol-*d*_4_) δ 8.09 (d, *J* = 8.0 Hz, 1H), 7.86 (d, *J* = 8.1 Hz, 1H); ^13^C NMR (126 MHz, methanol-*d*_4_) δ 166.3, 136.5, 136.0 (q, *J* = 32.6 Hz), 130.6, 127.0 (q, *J* = 4.0 Hz), 125.0 (q, *J* = 271.9 Hz), 121.0 (q, *J* = 321.5 Hz); ^19^F NMR (470 MHz, methanol-*d*_4_) δ −64.31 (s, 3F), −77.06 (s, 3F); HRMS (TOF) *m*/*z*: [M − H]^−^ calcd for C_9_H_4_F_6_NO_3_S^−^, 319.9822; found, 319.9869.

**Pentafluoro-*N*-((trifluoromethyl)sulfonyl)benzamide (9c):** Synthesized by general procedure. The product was obtained as clear needle-shaped crystal (2.40 g, 70%). mp 129–131°C; ^13^C NMR (126 MHz, methanol-*d*_4_) δ 158.6, 145.4 (dm, *J* = 257.0 Hz), 144.5 (dm, *J* = 256.8 Hz), 139.1 (dm, *J* = 250.0 Hz), 121.0 (q, *J* = 321.4 Hz), 111.9 (t, *J* = 17.6 Hz); ^19^F NMR (470 MHz, methanol-*d*_4_) δ −78.15 (s, 3F), −142.78 (d, *J* = 17.9 Hz, 2F), −152.25 (t, *J* = 19.2 Hz, 1F), −162.65 (t, *J* = 18.3 Hz, 2F); HRMS (TOF) *m*/*z*: [M − H]^−^ calcd for C_8_F_8_NO_3_S^−^, 341.9477; found, 341.9520.

**4-Bromo-*N*-((trifluoromethyl)sulfonyl)benzamide (9d):** Synthesized by general procedure. The product was obtained as white powder (2.98 g, 90%). mp 156–158 °C; ^1^H NMR (500 MHz, methanol-*d*_4_) δ 7.83 (d, *J* = 8.8 Hz, 2H), 7.73 (d, *J* = 8.7 Hz, 2H); ^13^C NMR (126 MHz, methanol-*d*_4_) δ 166.5, 133.3, 131.9, 131. 6, 129.9, 121.0 (q, *J* = 321.5 Hz); ^19^F NMR (470 MHz, MeOD) δ −77.00 (s); HRMS (TOF) *m*/*z*: [M − H]^−^ calcd for C_8_H_4_^79^BrF_3_NO_3_S^−^, 329.9053; found, 329.9095.

**Triethylamine salt of 4-bromo-*N*,*N*'-bis((trifluoromethyl)sulfonyl)benzimidamide (11):** 4-Bromo-*N*-((trifluoromethyl)sulfonyl)benzamide (**9d**, 1.68 g, 5.0 mmol) was mixed with POCl_3_ (3 mL) and PCl_5_ (1.11 g). The reaction mixture was stirred at rt for 2 h and then at 50 °C for 30 min. The solvent was removed and the imidoyl chloride was recrystallized from heptane (1.71 g, 98%). The imidoyl chloride (1.06 g) was dissolved in dry acetonitrile (4 mL) and the solution was added dropwise to a mixture of trifluoromethanesulfonamide (450 mg) and triethylamine (0.85 mL) in dry acetonitrile (8 mL) at 0 °C. The mixture was stirred at 0 °C for 30 min and then at rt for 5 h. The solvent was removed in vacuo and the residue was mixed with CH_2_Cl_2_ (30 mL) and 10% HCl (aq, 50 mL). The organic phase was separated and the water phase was extracted with CH_2_Cl_2_ (30 mL). The combined organic phase was washed with saturated NaHCO_3_ (aq) and dried with MgSO_4_. The solvent was removed in vacuo and the product was isolated as white powder (1.58 g, 93%). ^1^H NMR (500 MHz, methanol-*d*_4_) δ 7.73 (d, *J* = 8.7 Hz, 2H), 7.60 (d, *J* = 8.6 Hz, 2H), 3.20 (q, *J* = 7.3 Hz, 6H), 1.30 (t, *J* = 7.3 Hz, 9H); ^13^C NMR (126 MHz, methanol-*d*_4_) δ 170.0, 137.8, 132.3, 132.0, 127.6, 121.1 (q, *J* = 319.2 Hz), 48.0, 9.3; ^19^F NMR (470 MHz, MeOD) δ −80.67 (s); HRMS (TOF) *m*/*z*: [M − H]^−^ calcd for C_9_H_4_^79^BrF_6_N_2_O_4_S_2_^−^, 460.8706; found, 460.8769.

**4-Bromo-*N*,*N*'-bis((trifluoromethyl)sulfonyl)benzimidamide (12):** The trimethylamine salt of 4-bromo-*N*,*N*'-bis((trifluoromethyl)sulfonyl)benzimidamide (**11,** 0.50 g, 0.89 mmol) was mixed with H_2_SO_4_ (96%, 1.0 mL). The mixture was shaken for 30 min and extracted with CH_2_Cl_2_ (5 × 6 mL). The extract was dried with MgSO_4_ and the solvent removed in vacuo. The crude product was recrystallized from toluene and heptane to yield **12** as a white crystalline powder (367 mg, 0.79 mmol, 89%). ^1^H NMR (500 MHz, methanol-*d*_4_) δ 7.72 (d, *J* = 8.7 Hz, 2H), 7.59 (d, *J* = 8.6 Hz, 2H); ^13^C NMR (126 MHz, methanol-*d*_4_) δ 170.1, 137.6, 132.23, 131.9, 127.6, 121.02 (q, *J* = 319.3 Hz); ^19^F NMR (470 MHz, MeOD) δ −80.60 (s); HRMS (TOF) *m*/*z*: [M − H]^−^ calcd for C_9_H_4_^79^BrF_6_N_2_O_4_S_2_^−^, 460.8706; found. 460.8766.

**4-(1*H*-Benzo[*d*]imidazol-1-yl)-*N*-((trifluoromethyl)sulfonyl)benzamide (13):** Cesium carbonate (163 mg, 5 equiv) was added to a glass vial containing benzimidazole (17.7 mg, 1.5 equiv) and 4-fluoro-*N*-((trifluoromethyl)sulfonyl)benzamide (**9a**, 27.1 mg, 0.1 mmol) in dry dimethylacetamide (1.0 mL). The reaction was heated to 120 °C for 12 h. The reaction was quenched with 1.0 M HCl (aq, 100 μL) and the solvent removed in vacuo. The crude product was dissolved in acetonitrile/water 1:1 and filtered through a Teflon syringe filter. The solution was transferred to a C18 gel column and purified by vacuum liquid chromatography (VLC, 0 to 90% CH_3_CN in 0.01 M HCl). After lyophilization the product was obtained as a white powder (17 mg, 46%). ^1^H NMR (500 MHz, DMSO-*d*_6_) δ 9.64 (s, 1H), 8.20 (d, *J* = 8.5 Hz, 2H), 7.99–7.90 (m, 1H), 7.81 (d, *J* = 8.5 Hz, 3H), 7.64–7.53 (m, 2H), 4.53 (s, 2H); ^13^C NMR (126 MHz, DMSO-*d*_6_) δ 168.5, 142.3, 138.4, 136.0, 134.8, 131.4, 130.3, 126.0, 125.6, 124.0, 120.3 (q, *J* = 324.8 Hz), 116.6, 112.6; ^19^F NMR (470 MHz, DMSO-*d*_6_) δ −79.75 (s); HRMS (TOF) *m*/*z*: [M − H]^−^ calcd for C_15_H_9_F_3_N_3_O_3_S^−^, 368.0322; found, 368.0367.

**4-(1*H*-Benzo[*d*]imidazol-1-yl)-2,3,5,6-tetrafluoro-*N*-((trifluoromethyl)sulfonyl)benzamide (14):** Sodium *tert*-butoxide (21 mg, 2.2 equiv) was added to a glass vial containing benzimidazole (11.8 mg, 0.1 mmol) in dry dimethylacetamide (1.0 mL). The mixture was stirred at rt for 1 min. The mixture was cooled to 0 °C and added to a solution of **9c** (37.7 mg, 0.11 mmol) in dry dimethylacetamide (1.0 mL) under stirring at 0 °C. The reaction was allowed to reach rt and stirred for 1 h. The reaction was quenched with 1.0 M HCl (aq, 20 μL) and the solvent removed in vacuo. The crude product was dissolved in acetonitrile/water 1:1 and filtered through a Teflon syringe filter. The solution was transferred to a C18 gel column and purified by vacuum liquid chromatography (VLC, 0 to 90% CH_3_CN in 0.01 M HCl). After lyophilization the product was obtained as a white powder (30 mg, 81%). ^1^H NMR (500 MHz, DMSO-*d*_6_) δ 8.70 (s, 1H), 7.83 (dt, *J* = 7.3, 3.6 Hz, 1H), 7.66–7.57 (m, 1H), 7.47–7.34 (m, 2H); ^13^C NMR (126 MHz, DMSO) δ 161.34, 143.9, 143.6 (dddd, *J* = 246.1, 13.0, 8.4, 4.1 Hz), 142.2 (ddm, *J* = 252.7, 16.4 Hz), 141.3, 137.6 (dm, *J* = 254.1 Hz), 133.1, 124.5, 123.6, 121.4 (t, *J* = 21.9 Hz), 120.0 (q, *J* = 323.7 Hz), 119. 5, 114.1 (t, *J* = 14.6 Hz), 111.3; ^19^F NMR (470 MHz, DMSO-*d*_6_) δ −80.53 (s), −145.56 (dd, *J* = 25.2, 12.3 Hz), −148.81 (dd, *J* = 24.1, 11.3 Hz); HRMS (TOF) *m*/*z*: [M − H]^−^ calcd for C_15_H_5_F_7_N_3_O_3_S^−^, 439.9945; found, 434.0006.

**4'-Methoxy-*N*-((trifluoromethyl)sulfonyl)-[1,1'-biphenyl]-4-carboxamide (15):** Palladium(II) acetate (6 mg, 27 μmol), triphenylphosphine (20 mg, 76 μmol) and H_2_O (0.5 μL, 28 μmol) were mixed in degassed dimethylacetamide (1.0 mL). The catalyst was preformed by heating the mixture in a closed screw cap vial to 100 °C for 1 min (color changed from yellow to deep red). A part of the catalyst mixture (0.2 mL) was added to a microwave glass vial containing a degassed mixture of **9d** (33.1 mg, 0.1 mmol), 4-methoxyphenylboronic acid (22.8 mg, 0.15 mmol, 1.5 equiv) and cesium carbonate (100 mg, 3 equiv) in dimethylacetamide (0.8 mL). The vial was closed with a teflon cap and heated to 80 °C for 12 h under stirring. The reaction was quenched with 1.0 M HCl (aq, 100 μL) and the solvent removed in vacuo. The crude product was dissolved in acetonitrile/water 1:1 and filtered through a Teflon syringe filter. The solution was transferred to a C18 gel column and purified by vacuum liquid chromatography (VLC, 0 to 90% CH_3_CN in 0.01 M HCl). After lyophilization the product was obtained as a white powder (11 mg, 31%). ^1^H NMR (500 MHz, DMSO-*d*_6_) δ 7.96 (d, *J* = 8.3 Hz, 2H), 7.69–7.59 (m, 4H), 7.03 (d, *J* = 8.8 Hz, 2H), 3.80 (s, 3H); ^13^C NMR (126 MHz, DMSO-*d*_6_) δ 169.5, 159.2, 142.0, 135.7, 131.8, 129.2, 127.9, 125.4, 120.4 (q, *J* = 325.3 Hz). 114.4, 55.2; ^19^F NMR (470 MHz, DMSO-*d*_6_) δ −79.57 (s); HRMS (TOF) *m*/*z*: [M − H]^−^ calcd for C_15_H_5_F_7_N_3_O_3_S^−^, 358.0366; found, 358.0408.

## Supporting Information

File 1 NMR spectra of synthesized compounds.
