# Supplementary material for: Synthesis and stability of strongly acidic benzamide derivatives
Source: Beilstein J Org Chem. 2018 Feb 27;14:523–30. doi: 10.3762/bjoc.14.38 (PMC5852525; doi:10.3762/bjoc.14.38)

Supporting information

for

Synthesis and stability of strongly acidic benzamide derivatives

Frederik Diness [*, 1,2], Niels J. Bjerrum[3] and Mikael Begtrup[1]

Address: [1]Department of Drug Design and Pharmacology, University of Copenhagen, Universitetsparken 2, DK-2100 Copenhagen, Denmark, [2]Present address: Department of Chemistry, University of Copenhagen, Universitetsparken 5, DK-2100 Copenhagen, Denmark and [3]Department of Energy Conversion and Storage, Technical University of Denmark, Kemitorvet, Building 207, room 042, DK-2800 Kgs. Lyngby, Denmark

Email: Frederik Diness - fdi@chem.ku.dk

* Corresponding author

NMR spectra of synthesized compounds

4-Fluoro-*N*-((trifluoromethyl)sulfonyl)benzamide (**9a**).

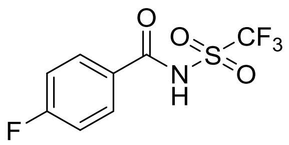

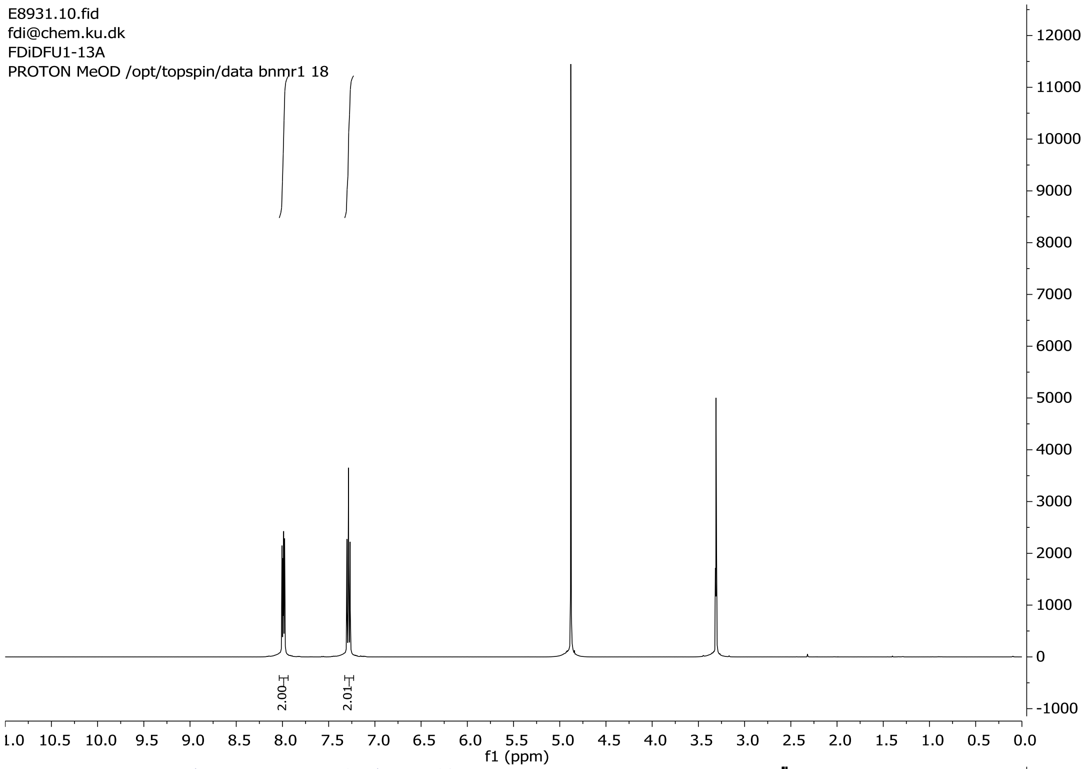

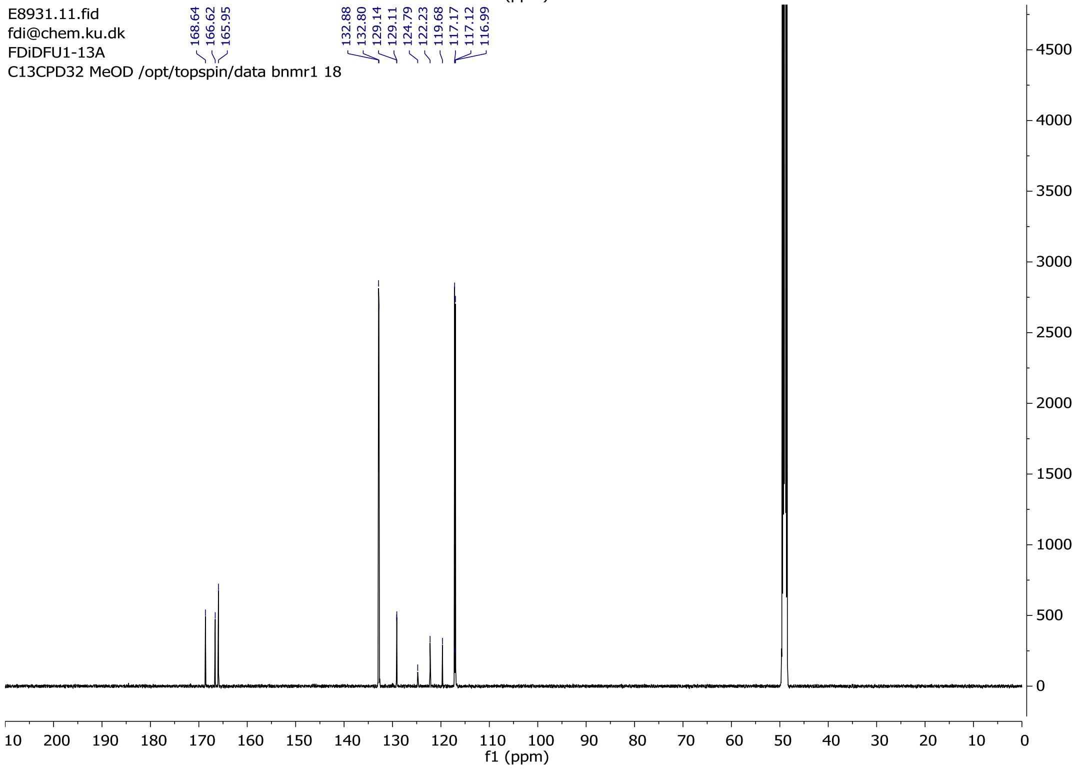

4-Trifluoromethyl-*N*-((trifluoromethyl)sulfonyl)benzamide (**9b**).

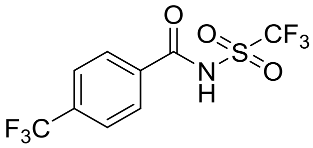

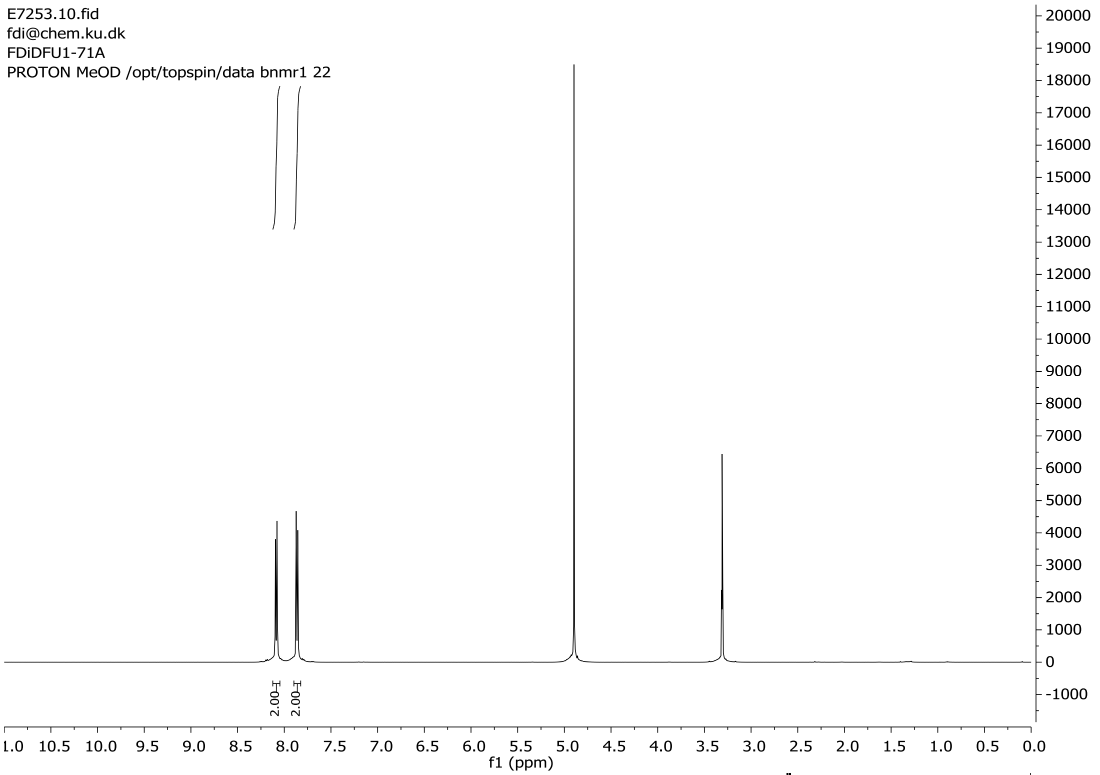

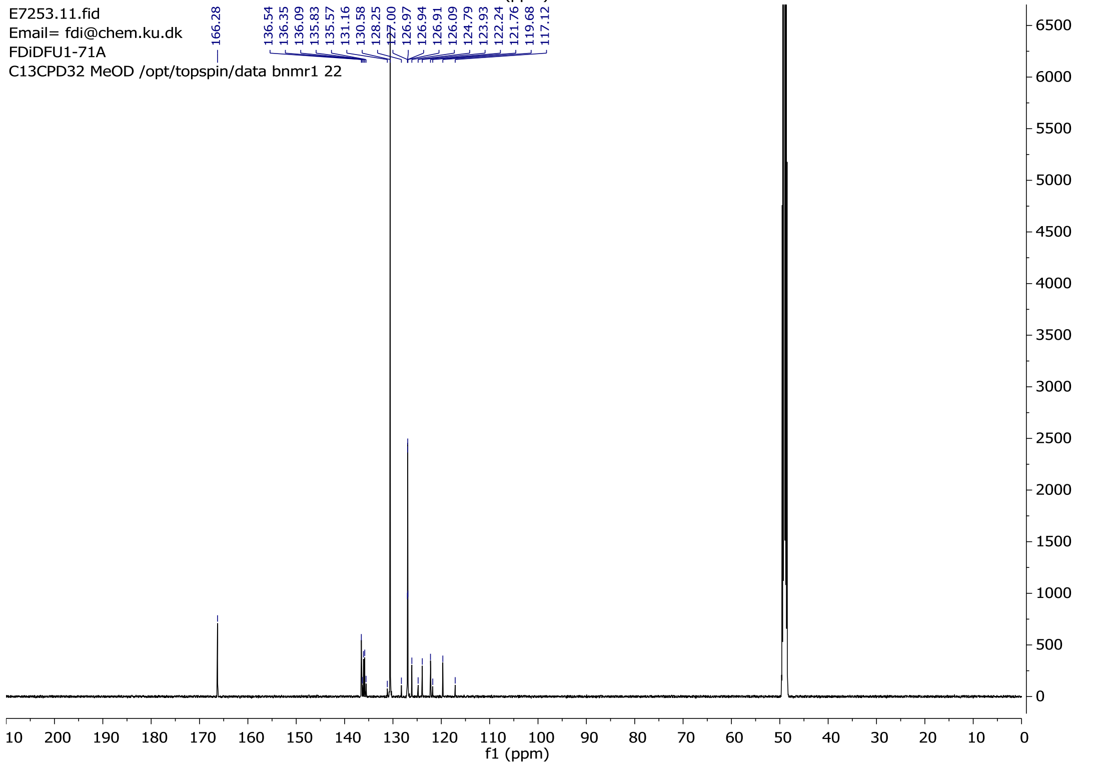

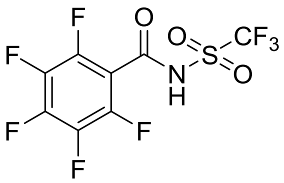

Pentafluoro-*N*-((trifluoromethyl)sulfonyl)benzamide (**9c**).

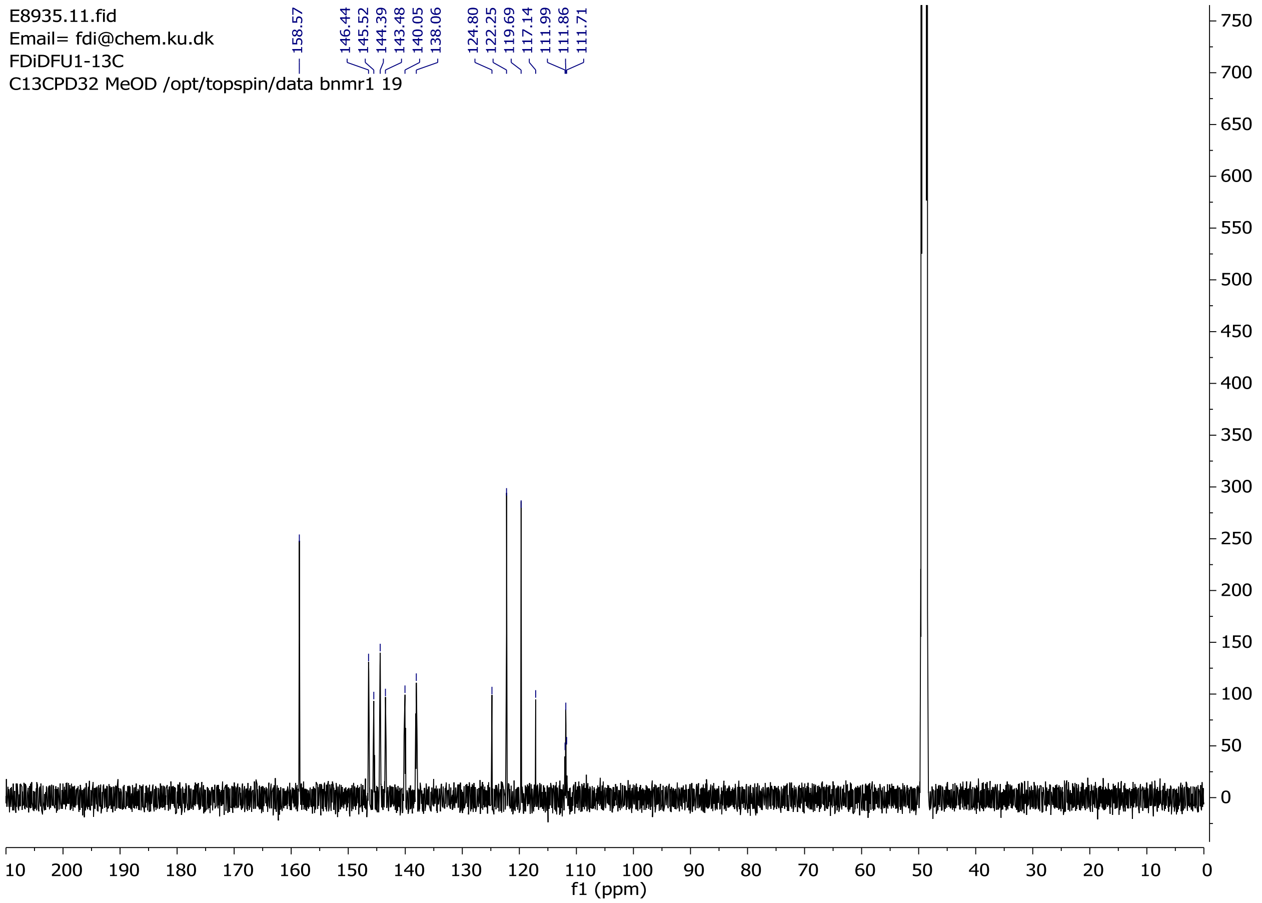

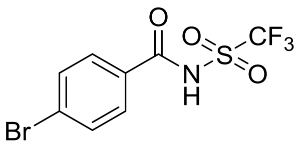

4-Bromo-*N*-((trifluoromethyl)sulfonyl)benzamide (**9d**).

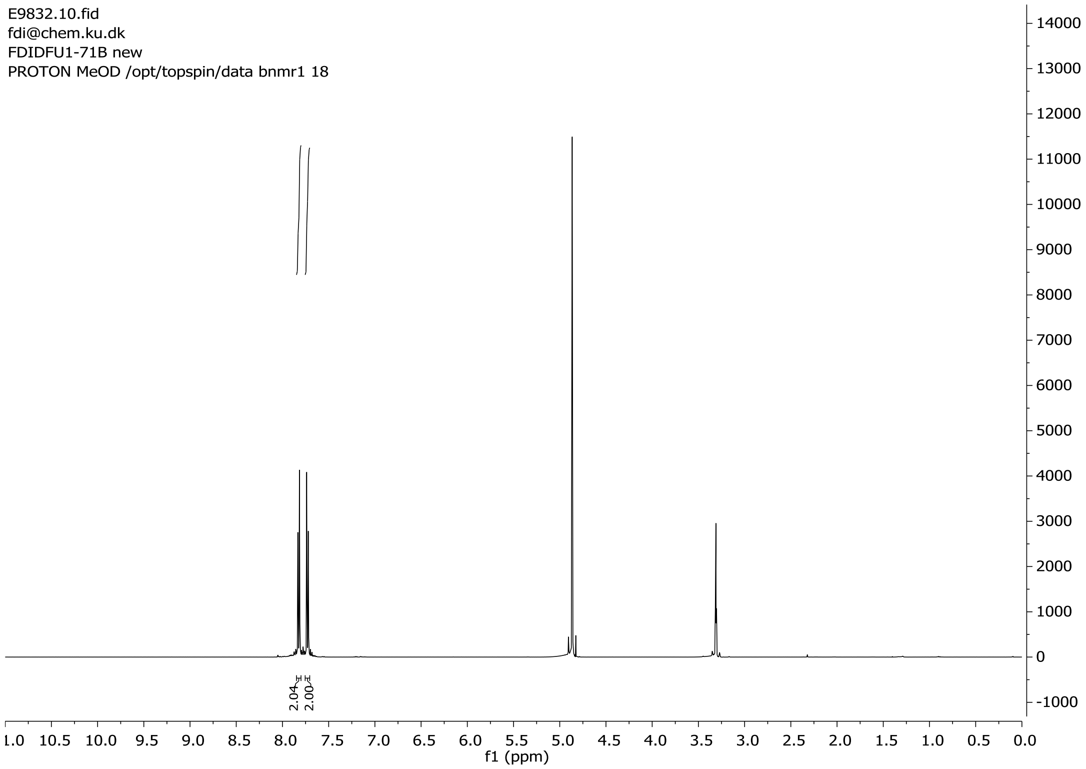

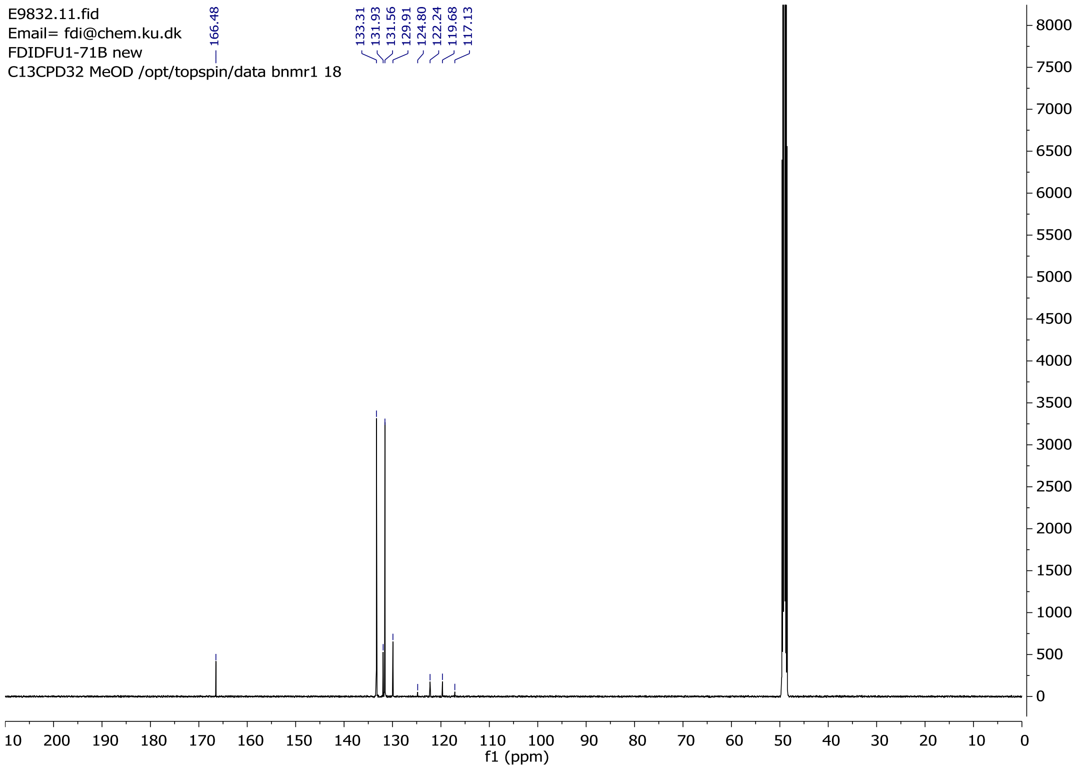

Triethylamine salt of 4-bromo-*N*,*N'*-bis((trifluoromethyl)sulfonyl)benzimidamide (**11**).

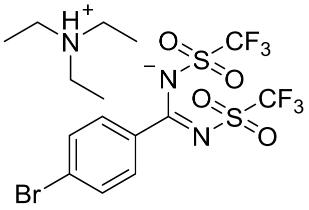

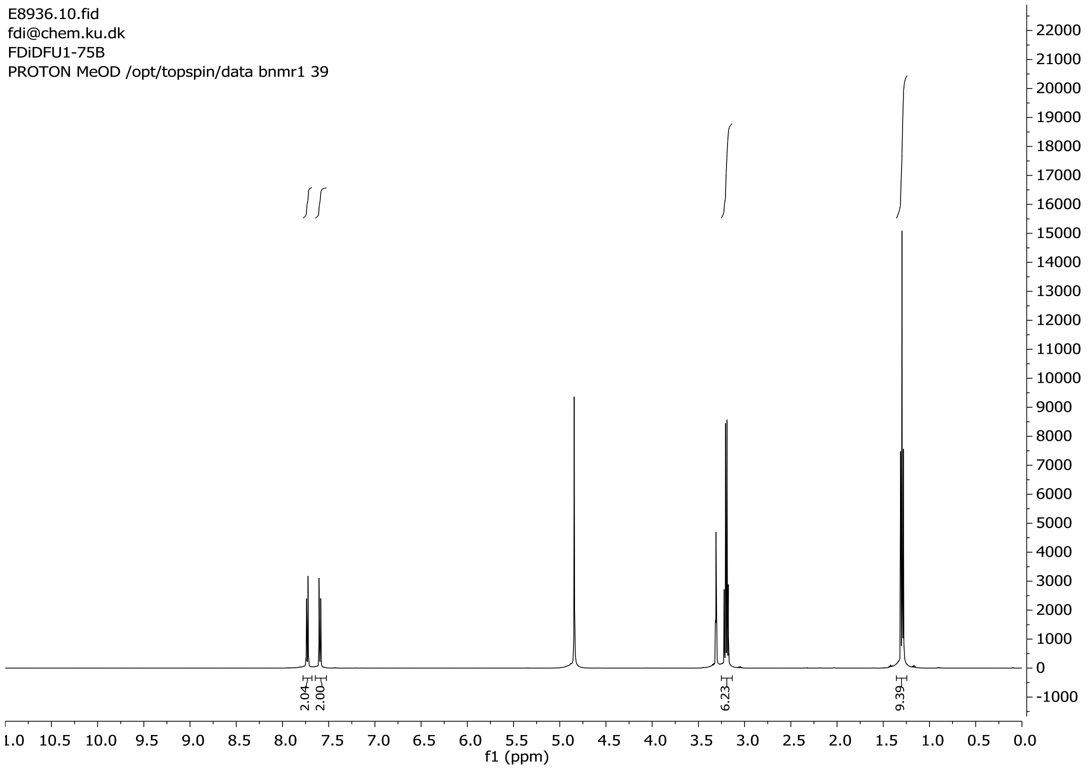

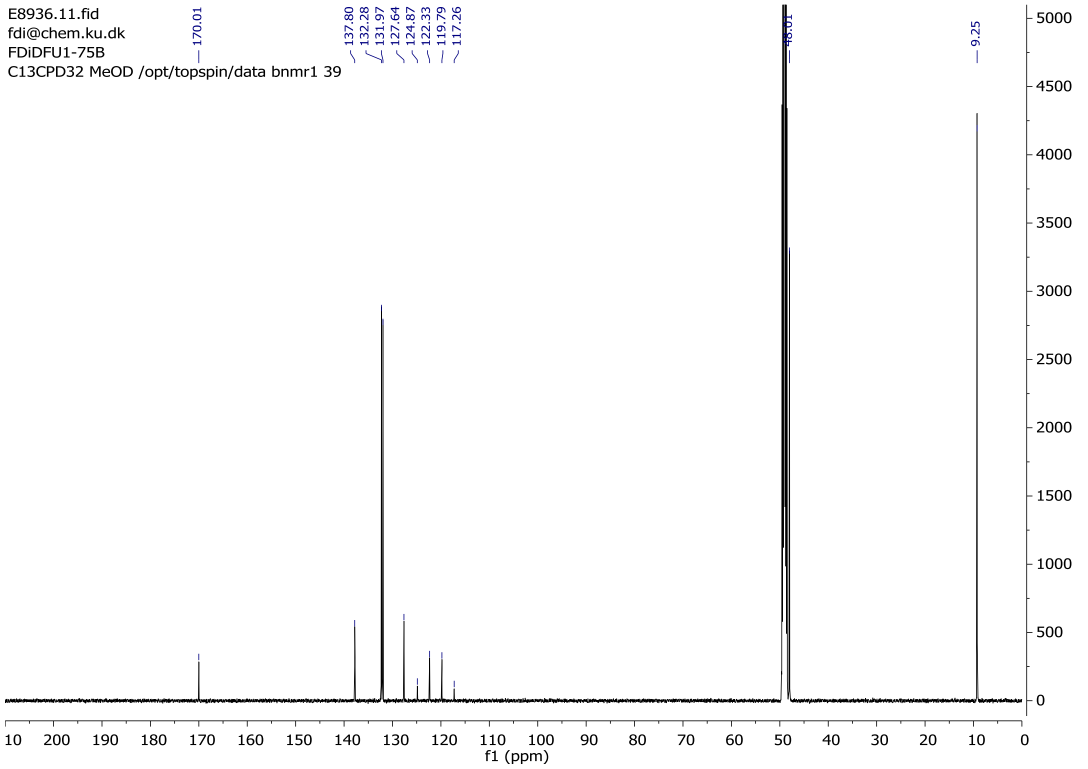

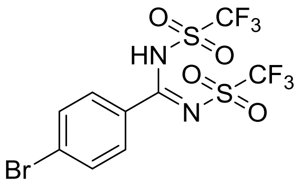

4-Bromo-*N*,*N'*-bis((trifluoromethyl)sulfonyl)benzimidamide (**12**).

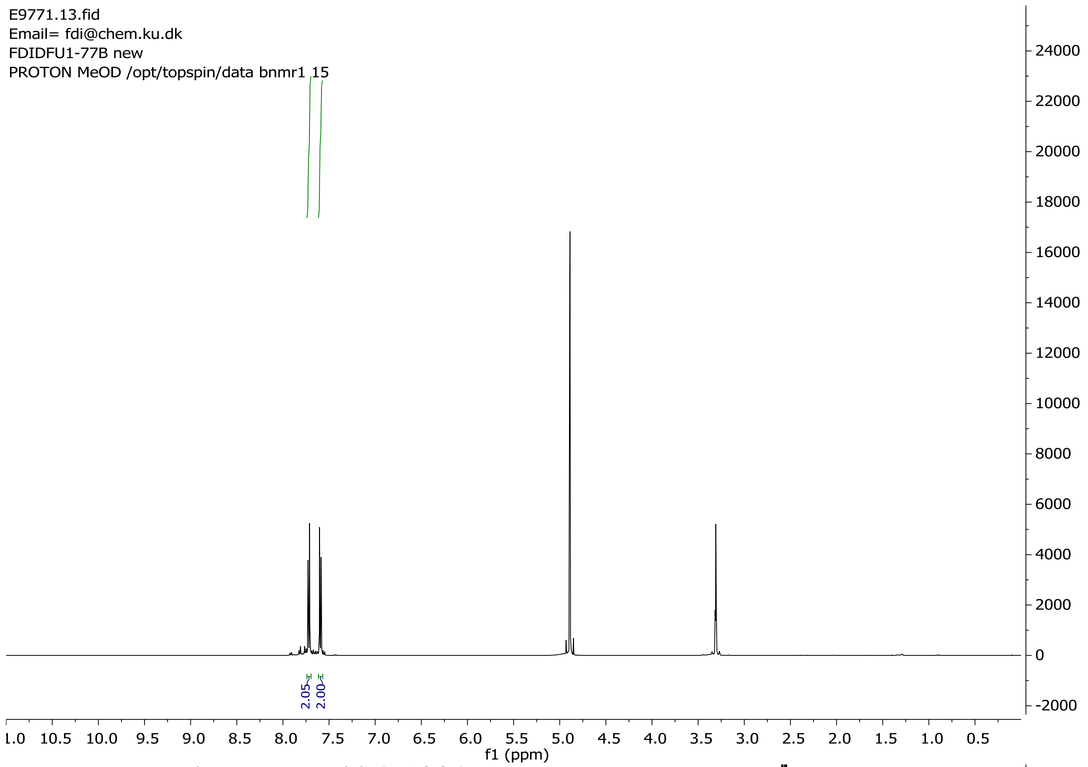

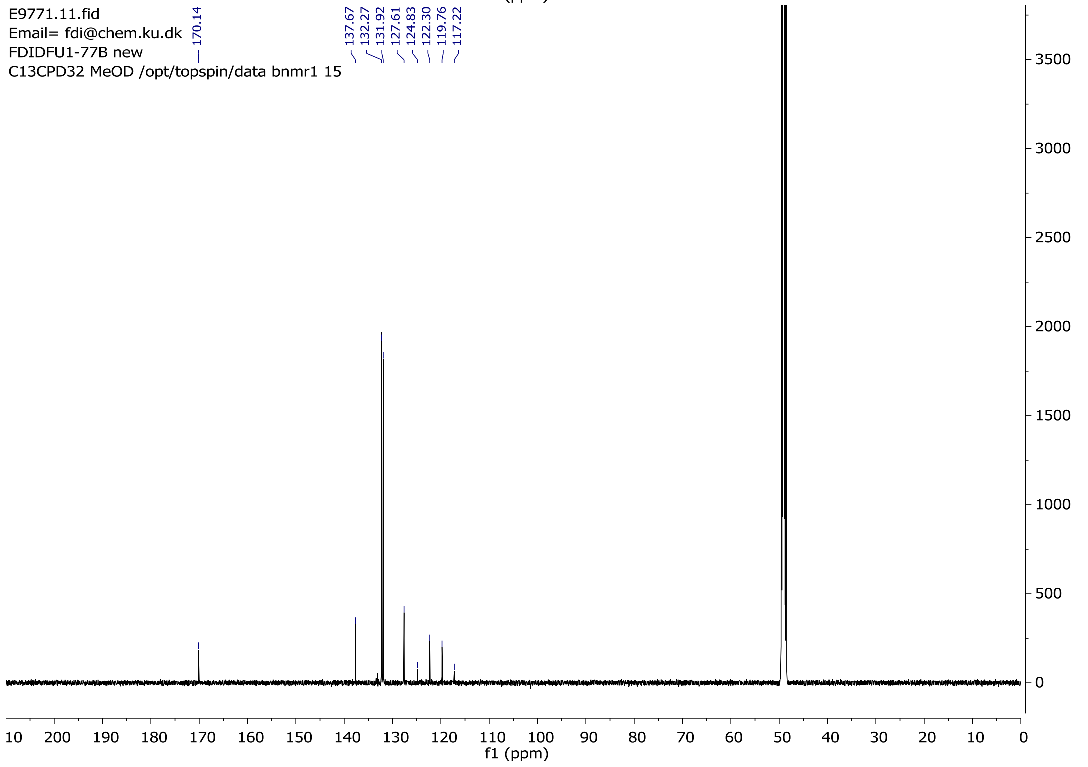

4-(1*H*-Benzo[d]imidazol-1-yl)-*N*-((trifluoromethyl)sulfonyl)benzamide (**13**).

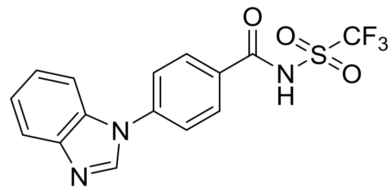

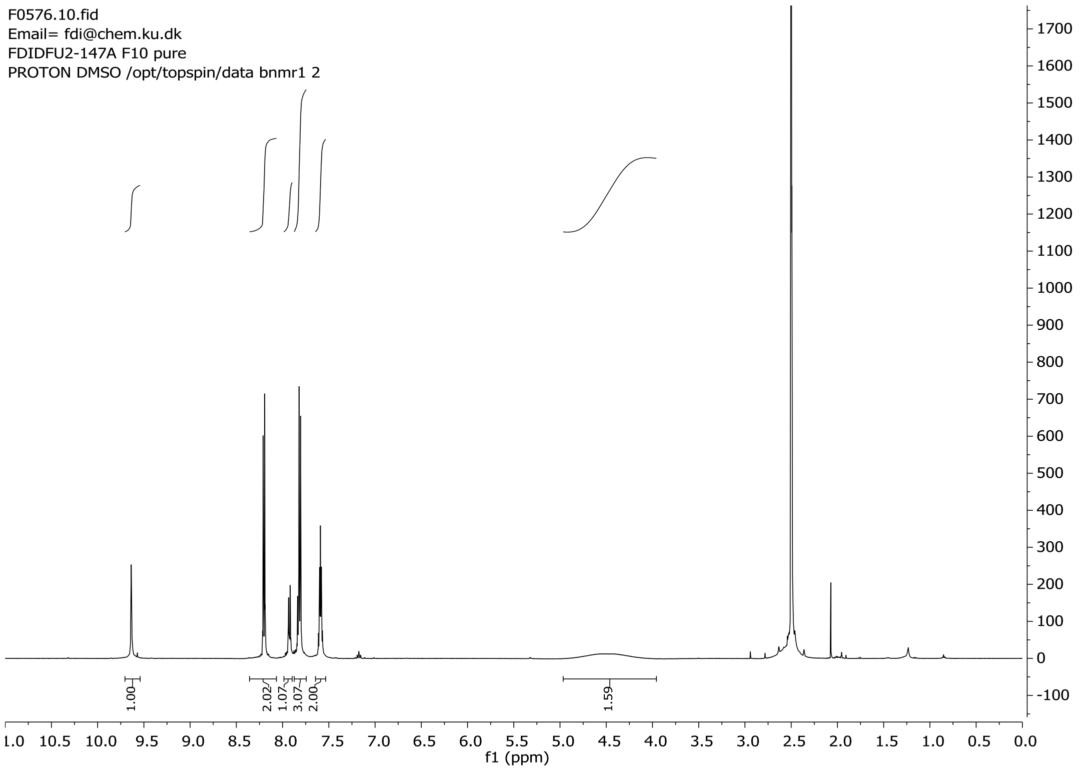

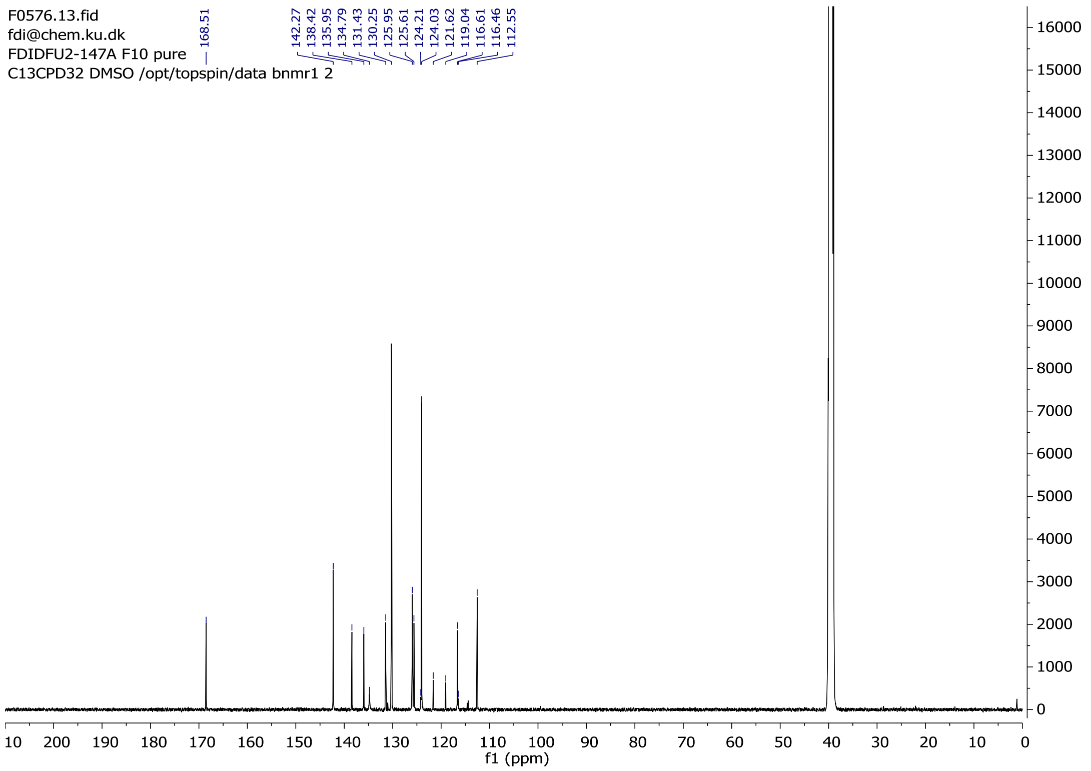

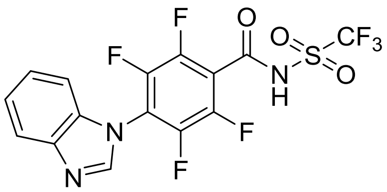

4-(1*H*-Benzo[d]imidazol-1-yl)-2,3,5,6-tetrafluoro-*N*-((trifluoromethyl)sulfonyl)benzamide (**14**).

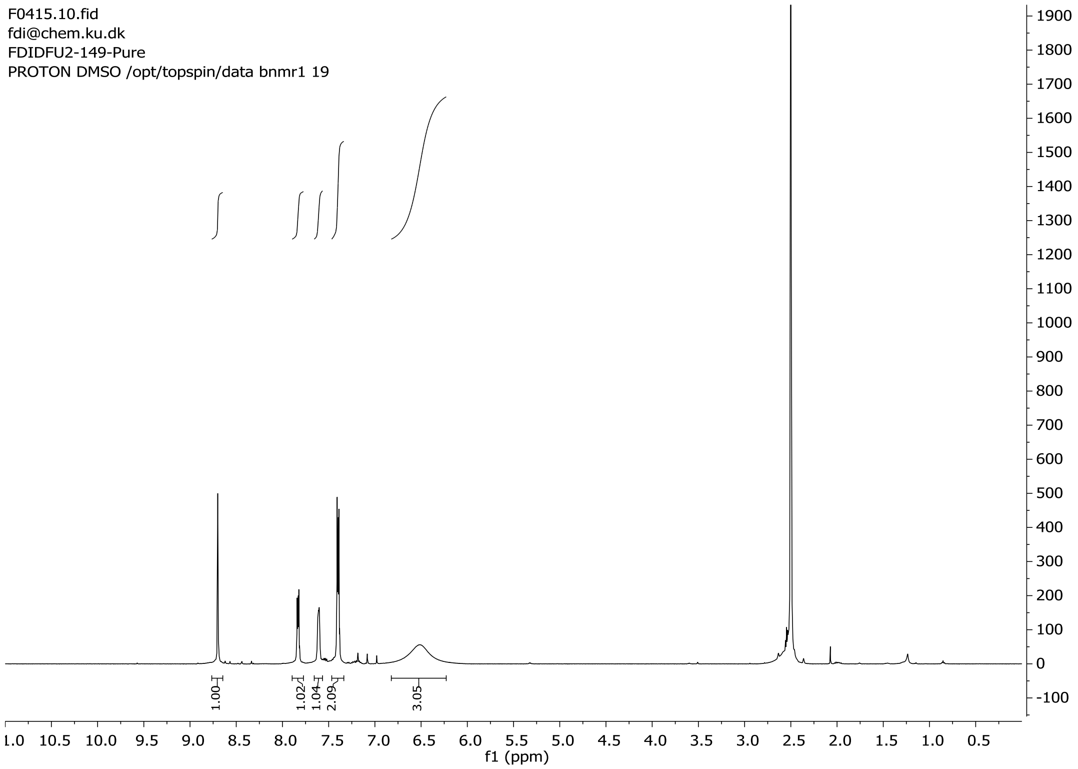

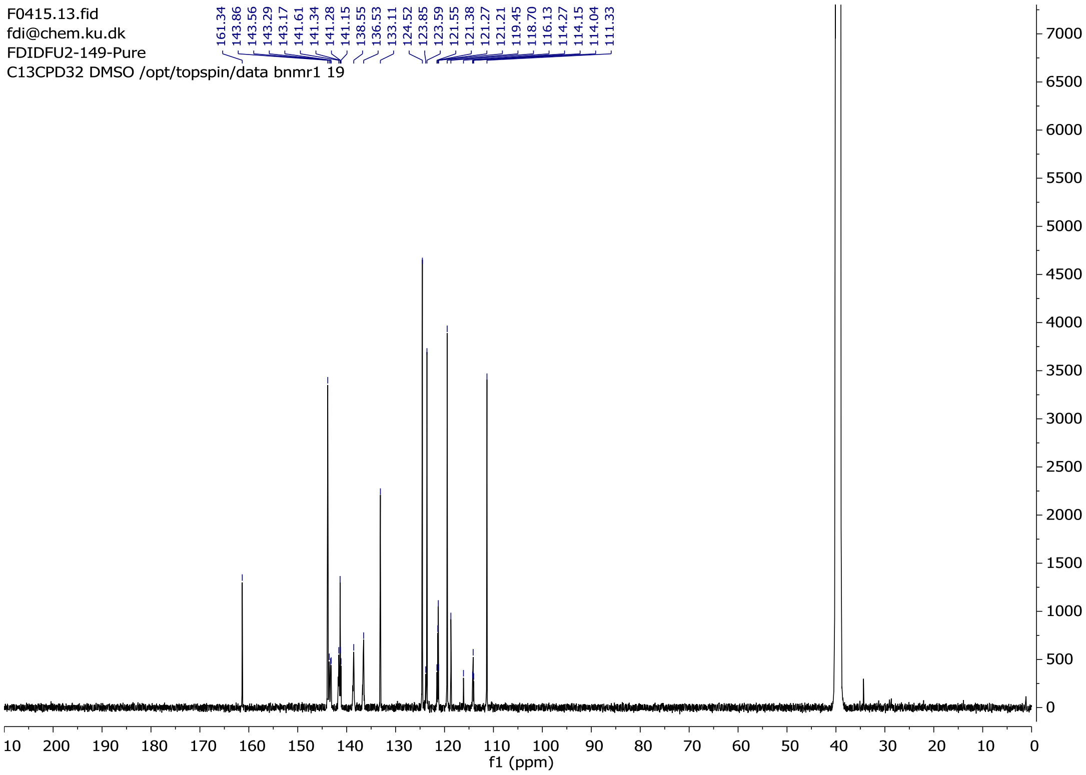

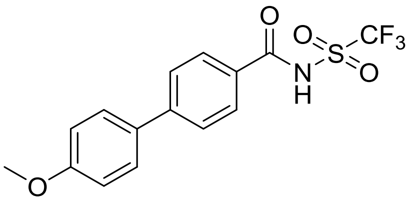

4'-Methoxy-*N*-((trifluoromethyl)sulfonyl)-[1,1'-biphenyl]-4-carboxamide (**15**).

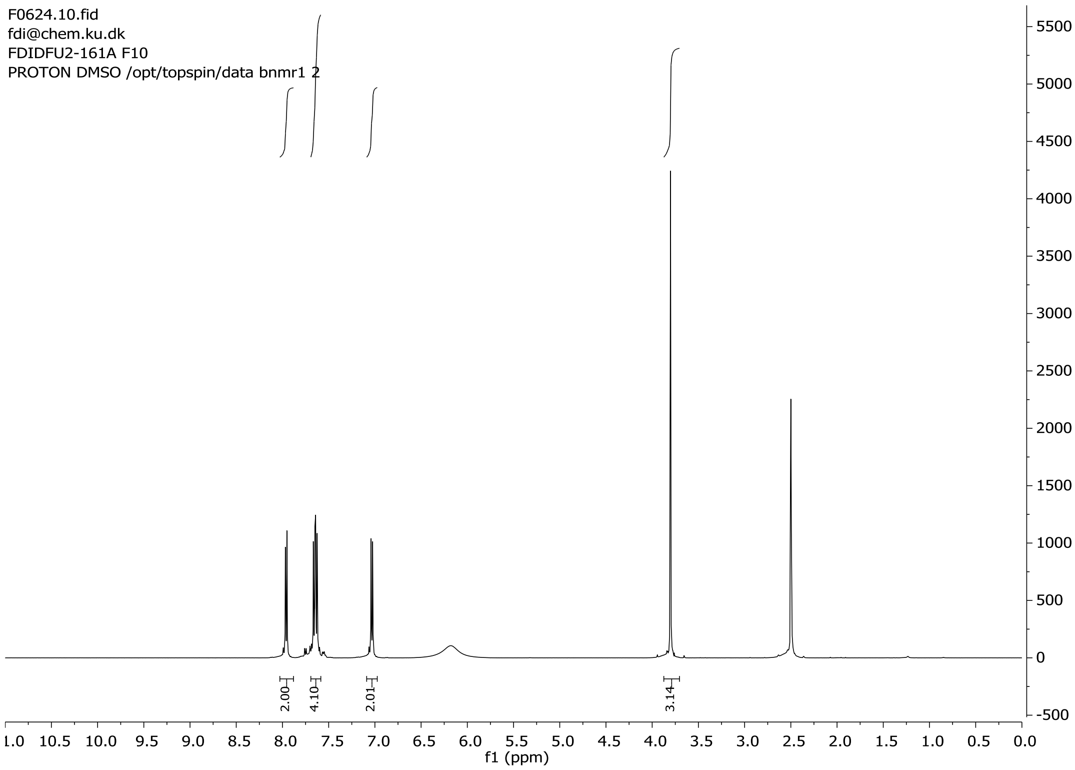

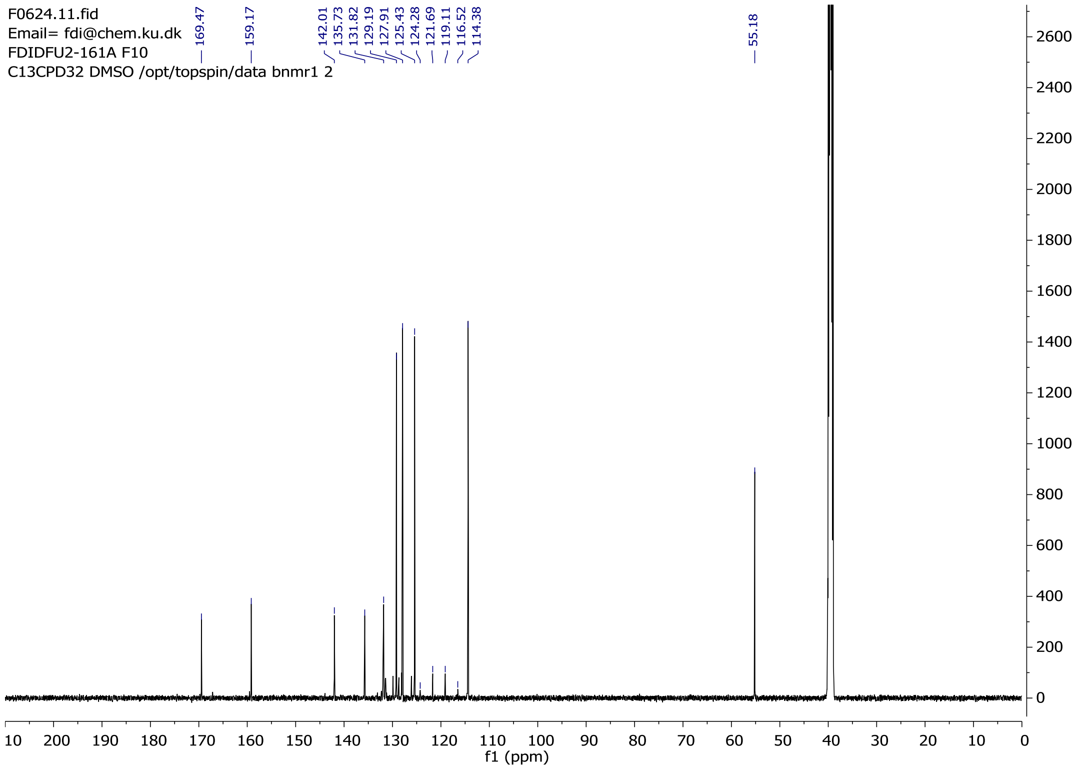

Supplement: File 1 — NMR spectra of synthesized compounds. [file Beilstein_J_Org_Chem-14-523-s001.pdf]
